# Data on the ^14^C date obtained from the charcoal figure “Black fox” in Shulgan-Tash (Kapova) cave, Southern Ural, Russia

**DOI:** 10.1016/j.dib.2018.10.040

**Published:** 2018-10-17

**Authors:** Yuri Dublyansky, Yuri Lyakhnitsky, Christoph Spötl

**Affiliations:** a Institute of Geology, Innsbruck University, Innrain 52, Innsbruck 6020, Austria; b A.P. Karpinsky Russian Geological Research Institute (VSEGEI), Sredny prospekt 74, St.-Petersburg 199106, Russian Federation

**Keywords:** Shulgan-Tash cave, Radiocarbon, Cave art

## Abstract

Shulgan-Tash (Kapova) cave in southern Ural, Russia, is the easternmost European site hosting Late Paleolithic cave art. Most of the 195+ drawings catalogued in the cave are made with red natural pigment (ochre), and only a handful of drawings are made with charcoal, see “Catalogue of images” [4], “Höhlenmalerei im Ural: Kapova und Ignatievka; die altsteinzeitlichen Bilderhöhlen im sudlichen Ural,” [5]. “Constraining the ages of the Late Palaeolithic cave paintings in the Shulgan-Tash cave, Southern Urals, Russia” [6]. None of the charcoal drawings were directly dated by ^14^C method so far.

Black lines delineating a figure similar to the outline of a fox are known in the cave. Here we present data on the ^14^C AMS date of charcoal with which the lines were drawn. Calibration of the data was performed using the Bomb13NH1 dataset, see “Atmospheric radiocarbon for the period 1950–2010” [7] and the IntCal13 dataset, see “IntCal13 and Marine13 radiocarbon age calibration curves 0–50,000 years cal BP” [8]. The calibrated age distribution has maximum probability density (65.3%) between 1877 and 1918.

**Specifications table**

**Table:** 

Subject area	*Archaeological science*
More specific subject area	*Geochronology, history, archaeology*
Type of data	*Tables, photo images, graph*
How data was acquired	*The acceleration mass spectrometry (AMS) system at Oxford Radiocarbon Accelerator Unit (University of Oxford, UK)*
Data format	*Raw and analyzed*
Experimental factors	*Samples were treated with acid, base, acid sequence as per*[1], [2], [3].
Experimental features	*Samples were combusted in SHN analyzer furnace system, passed through a gas chromatograph and measured for δ*^*13*^*C values. An aliquot of CO*_*2*_*collected from the He stream was mixed with hydrogen, reduced to graphite on an Fe catalyst and pressed into a target for the ion source as per*[1], [2], [3].
Data source location	*Shulgan-Tash (Kapova) cave; 53°2′34.80″N; 57°3′57.60″E; Russia*
Data accessibility	*Data is reported in this article.*

**Value of the data**

• The data can be used by scientific community to constrain the age of charcoal of the lines, creating the figure “Black fox” in Shulgan-Tash (Kapova) cave, Russia.
• This is the first direct radiocarbon dating of a charcoal drawing in Shulgan-Tash cave can be used by archaeologists and historians studying the use of the cave by humans in the past.
• The data can be used to close the controversy as to whether this particular figure is of Paleolithic age or is a later artifact.

## 1 Data

**Fig. 1:**
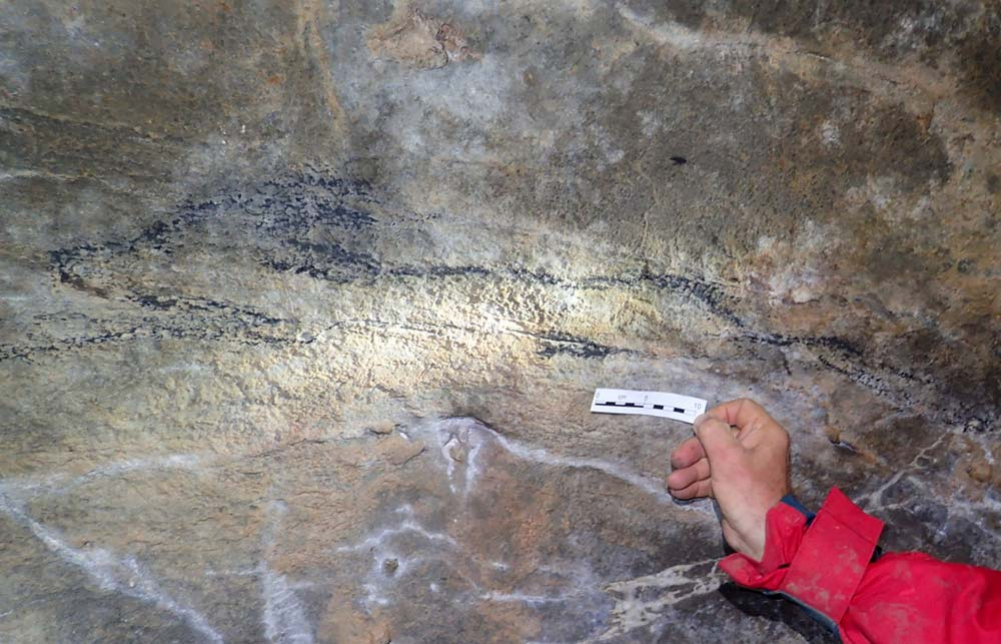
The “Black fox” figure on the ceiling of a niche in the western wall of the Hall of Drawings, Shulgan-Tash cave, Russia. Scale is 10 cm.

**Fig. 2:**
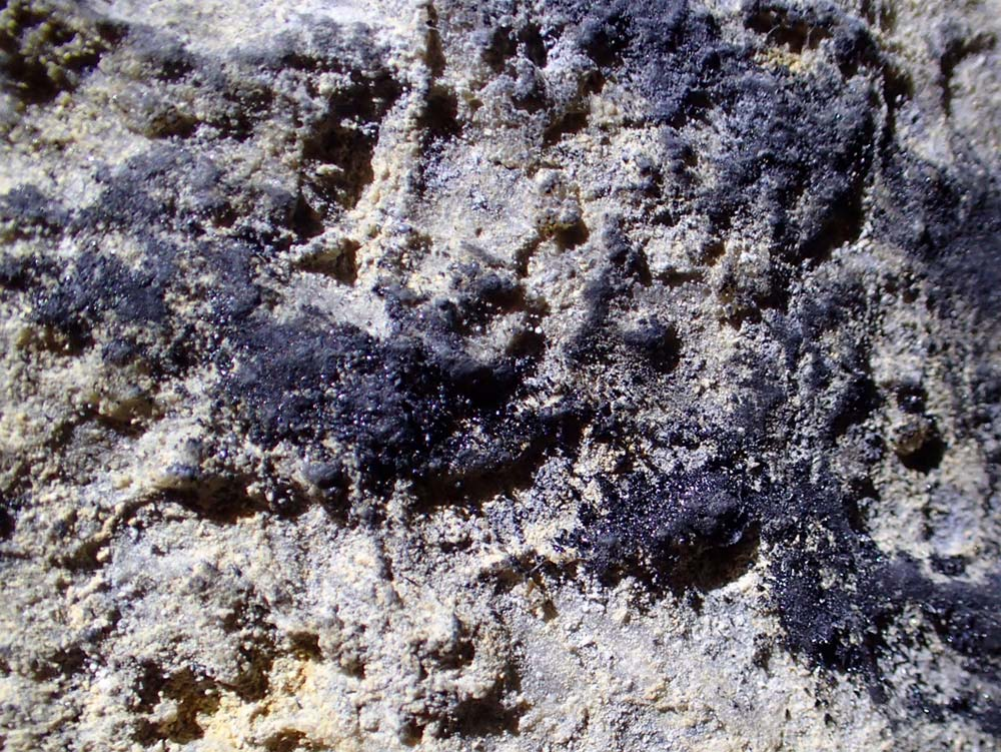
Close-up on the lines. Charcoal from these lines was collected for ^14^C dating. Width of frame is ca. 5 mm.

Black charcoal lines, delineating a figure evocative of a running fox (Fig. 1) were sampled in the Hall of Drawings of the Shulgan-Tash (Kapova) cave in southern Ural, Russia. The length of the figure is 84 cm; its width is 18 cm. In contrast to several other charcoal drawings found in the cave, which are poorly preserved, faint and barely visible (and, therefore, not amenable to radiocarbon dating [4], [5]) the lines composing the figure are sharp and bright, with discrete particles of charcoal clearly visible (Fig. 2). The figure was identified as a drawing, and assigned the name “Black fox” in Ref. [4 p. 94].

**Fig. 3:**
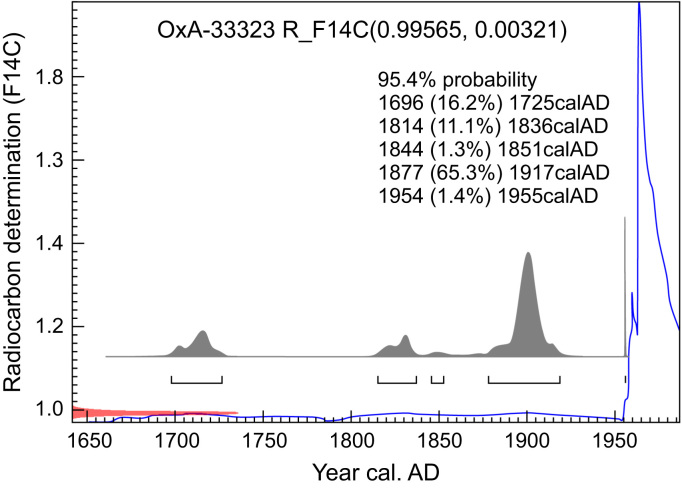
Probability age distribution of a charcoal sample from the figure of “Black fox” in Shulgan-Tash cave (Russia) calculated using OxCal v4.3.2 ©Bronk Ramsey (2017). Blue line is the NH1 curve of [8].

The data presented in this paper consist of photo documentation of the sample collection site (Figs. 1 and 2), the ^14^C (AMS) dating results (Table 1), the results of calibration of the ^14^C date (Table 2), and graphical representation of obtained dates (Fig. 3).

**Table 1:** Results of ^14^C dating.

**Sample ID**	**Provenance**	**Material**	**δ**^**13**^**C (‰ VPDB)**	**F**^**14**^**С**	^**14**^**C date (yrs BP)**
OxA-33323	Shulgan-Tash cave	charcoal	−27.32	0.99565 ± 0.00321	35 ± 26

Note: BP = Before present – AD 1950.

**Table 2:** Calibrated ^14^C date of charcoal.

**From (yrs AD)**	**To (yrs AD)**	**Probability (%)**
*68.2% probability*		
1710	1718	7.9%
1828	1832	3.6%
1890	1910	55.4%
1955	1955	1.3%
*95.4% probability*		
1696	1725	16.2%
1814	1836	11.1%
1844	1851	1.3%
1877	1917	65.3%
1954	1955	1.4%

The data was calibrated using OxCal (v4.3.2; © Bronk Ramsey 2017) using the Bomb13NH1 curve [7], which is valid for latitudes north of 40°N and is prepended with the last 300 years of the IntCal13 dataset [8]. Calibrated dates and their probabilities are shown in Fig. 3 and Table 2.

## 2 Experimental design, materials, and methods

A sample was collected by chipping small grains of charcoal from different parts of the drawing with a scalpel. This technique allowed collection of sufficient amount of material without affecting the visual appearance of the drawing.

The sample was analyzed in the Research Laboratory for Archaeology and the History of Art, University of Oxford (UK). Preparation of the sample (id:OxA-33323) and analysis by Acceleration Mass Spectrometry (AMS) was performed according to protocols and procedures reported in Refs. [1], [2], [3]. The measured radiocarbon age was corrected for stable isotope fractionation using the measured value δ^13^C = −27.3‰ VPDB.
